# Surface electronic corrugation of a one-dimensional topological metal: Bi(114)

**DOI:** 10.1039/d1cp05284e

**Published:** 2022-02-09

**Authors:** Stephan J. Schmutzler, Adrian Ruckhofer, Wolfgang E. Ernst, Anton Tamtögl

**Affiliations:** Institute of Experimental Physics, Graz University of Technology 8010 Graz Austria ruckhofer@tugraz.at tamtoegl@gmail.com; Freie Universität Berlin, Fachbereich Physik Arnimallee 14 14195 Berlin Germany

## Abstract

The surface of Bi(114) is a striking example where the reduced dimensionality gives rise to structural rearrangement and new states at the surface. Here, we present a study of the surface structure and electronic corrugation of this quasi one-dimensional topological metal based on helium atom scattering (HAS) measurements. In contrast to low-index metal surfaces, upon scattering from the stepped (114) truncation of Bi, a large proportion of the incident beam is scattered into higher order diffraction channels which in combination with the large surface unit cell makes an analysis challenging. The surface electronic corrugation of Bi(114) is determined, using measurements upon scattering normal to the steps, together with quantum mechanical scattering calculations. Therefore, minimisation routines that vary the shape of the corrugation are employed, in order to minimise the deviation between the calculations and experimental scans. Furthermore, we illustrate that quantum mechanical scattering calculations can be used to determine the orientation of the in- and outgoing beam with respect to the stepped surface structure.

## Abstract 2

We present helium atom scattering measurements, normal to the steps of vicinal Bi(114), together with quantum mechanical scattering calculations. We thus obtain a representation of the surface electronic corrugation of the quasi one-dimensional metal.

## Graphical abstract

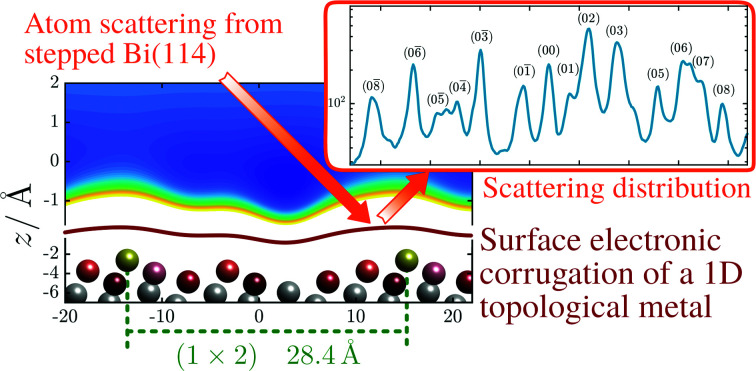

## 1 Introduction

An attractive path to study systems of reduced dimensionality is to create them on the surfaces of semiconducting or semimetallic substrates. Many systems have been realised and studied in this way recently, such as metallic chains or graphene nanoribbons on semiconductors.^1^ In fact, such systems with reduced dimensionality, have often been the key to the discovery of fundamentally new physics. The formation of the surface itself may give rise to drastic changes, *e.g.*, for polar semiconductors faceting is expected and unique surface orientations forming low-energy vicinal surfaces have been observed, in order to account for the unstable bulk termination.^2^

The semimetal surfaces of Bi and Sb are prominent examples of materials where the physical and chemical properties are radically different from those of the corresponding bulk material. *E.g.* in both materials surface electronic states exist^3–5^ and in the case of Sb(111), charge density waves have been observed.^6^ Both Bi and Sb are also elemental building blocks of binary topological insulators with their unique electronic structure which exhibits a protected conducting surface as well as insulating bulk states.^5,7–14^ More importantly, stepped surfaces are particularly interesting for the realisation of quasi one-dimensional systems^15^ and they provide an ideal playground to study *e.g.* site-specific catalytic reactivity^16–18^ or ice formation at a highly corrugated adsorption template.^19^

**Fig. 1:**
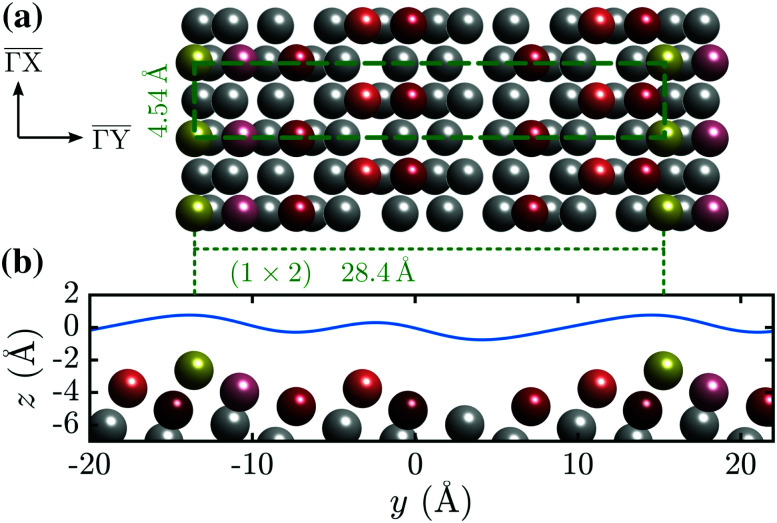
(a) Top view of the (1 × 2) reconstructed Bi(114) surface. (b) Side view of the atomic structure of Bi(114) surface along the 
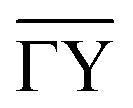
-direction, illustrating the asymmetry upon scattering normal to the steps. An exemplary corrugation function (eqn (3)) using *j* = 3 terms with *h* = [0.435,0.43,0.14] and 
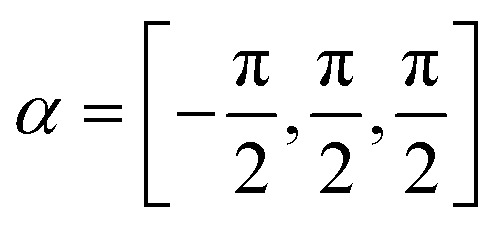
 is drawn as solid blue line above the side view of the atomic structure.

In this work we describe a study of the stepped (114) truncation of Bi (Fig. 1), which is a quasi-one dimensional topological metal.^20^ The surface undergoes a (1 × 2) reconstruction at room temperature resulting in a unit cell length of 28.4 Å normal to the steps (
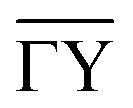
 in Fig. 1(a)). Such a large unit cell makes any detailed quantitative analysis and modelling approaches particularly challenging. We rely on scattering experiments which are strictly surface sensitive and in addition to the surface structure provide information about the surface electronic corrugation and the atom-surface interaction potential.^21–23^ The latter are necessary prerequisites for any quantitative description and theoretical treatment of molecular adsorption,^24^ scattering approaches to chemisorption^25,26^ and the coordinates relevant to the reaction potential^27–29^ within the wide aspects of physical chemistry. We provide an in-depth analysis of the experimentally measured scattering intensities based on quantum-mechanical scattering calculations.

## 2 Experimental and computational details

### 2.1 Experimental details

The Bi(114) crystal was cleaned by repeated circles of Ar^+^-sputtering and annealing to 400 K until clear diffraction peaks during helium atom scattering (HAS) were observed. The HAS measurements were performed by scattering a nearly monochromatic beam of He (Δ*E*/*E* ≈ 2%) off the sample surface in a fixed 91.5° source-sample-detector geometry (for a detailed description refer to ref. 30). The angular diffraction scans (*ϑ*-scans) were produced by rotating the sample in the scattering plane to yield different incident angles. The diffraction scans were performed either at room temperature (300 K) or at cryogenic temperatures (113 K) *via* a thermal connection to a liquid nitrogen reservoir.

#### A quasi one-dimensional metal: Bi(114)

As mentioned above, the studied (114) truncation of Bi, undergoes a surface reconstruction at room temperature and is considered a quasi one-dimensional topological metal.^14,20^ It further supports a dimerisation and charge density wave along the rows at low temperature.^1,31^Fig. 1 shows the top and side view of the (1 × 2) reconstructed Bi(114) surface with a very large lattice vector of 28.4 Å in the 
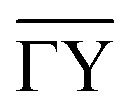
-direction. The side view along 
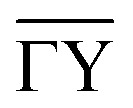
 (Fig. 1(b)) reveals the stepped structure of the (114) truncation with an additional step (protrusion) within the unit cell.

**Fig. 2:**
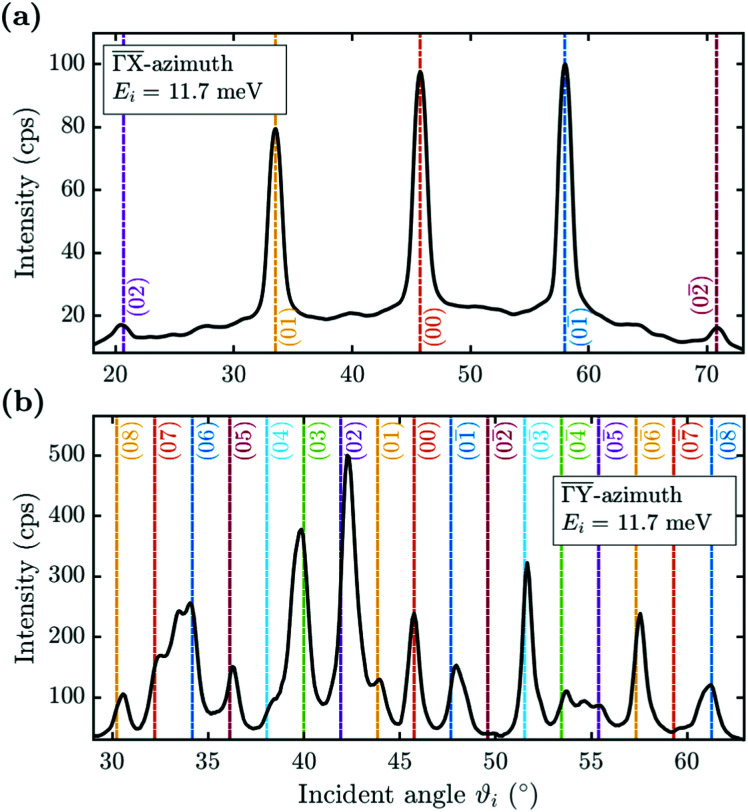
Diffraction scans along the two high-symmetry directions upon scattering parallel (
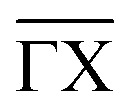
 (a)) and normal (
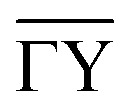
 (b)) to the steps, plotted *versus* incident angle *ϑ*_*i*_ at a sample temperature of *T*_S_ = 113 K. The individual diffraction peaks are labelled with the interacting **G**-vector.

He scattering occurs from the surface electronic corrugation above the ion cores,^21,32,33^ as illustrated by the exemplary corrugation in Fig. 1(b). Elastic diffraction scans parallel to the steps give rise to a diffraction scan similar to the one obtained for the Bi(111) surface^34^ – as shown in Fig. 2(a) for the 
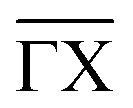
-azimuth with the specular reflection at 45.75°. The individual diffraction peaks are labelled with the respective interacting **G**-vector. Additional small features in between the main diffraction peaks arise due to a dimerisation at low temperatures.^1,31^ This (2 × 2) reconstruction is caused by the 
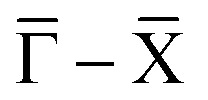
 intervalley electron–phonon coupling.^35^ From the temperature dependence of the Debye–Waller exponent of the specular intensity in 
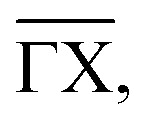
 theoretical calculations determined the electron–phonon coupling constant to be *λ* = 0.45 when treating the system as a 1D free-electron gas.^35^

Scattering normal to the steps 
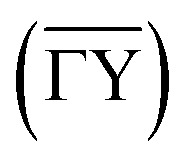
 as shown in Fig. 2(b) on the other hand, gives rise to a multitude of diffraction peaks due to the large unit cell. Diffraction peaks up to 8th order were resolved at incident energies between *E*_*i*_ = 10–15 meV. It is noteworthy that in Fig. 2(b), some diffraction peak positions do not exactly coincide with the calculated **G**-vector positions. A possible explanation is the effect of the the large unit cell in combination with the kinematic and angular broadening of the individual peaks, causing some of the maxima to be obscured by the shoulders of the next-nearest peaks. Additionally, selective adsorption resonances may also cause additional peaks. Due to the large “asymmetric” corrugation along 
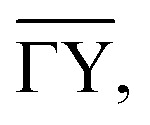
 the diffraction scans are not symmetric around the specular reflection either. Moreover, the highest intensity is typically scattered into a different diffraction channel than the (00) channel, analogous to scattering form a blazed grating in optics.^36,37^ While higher ratios of scattered intensity between nonzeroth-order diffraction and the specular peak have been demonstrated to be correlated with increased surface corrugation alone,^38^ the emerging beam resonance effect upon scattering of atomic and molecular beams from blazed gratings has been studied by Schöllkopf and coworkers.^39^ For the latter effect it was shown that the ratio of intensities scattered into specific diffraction channels depends strongly on the details of the particle surface interaction, based on quantum mechanical scattering calculations.^40^

### 2.2 Computational details

#### Quantum-mechanical scattering calculations

Certain properties of a sample are difficult to measure directly, therefore indirect methods can be used. Following quantum mechanical scattering calculations the intensities in the angular diffraction scans can be determined and compared with the experimental intensities.^21^ For HAS measurements, an established method is the so-called close-coupling (CC) approach.^41,42^ As the heights of the diffraction peaks contain information about the physical interaction between the impinging particles and the surface, this information becomes accessible when the measured data is sufficiently well reproduced by the simulation.

Starting point for the elastic CC approach is the time-independent Schrödinger equation, where upon insertion of a Fourier series expansion of the surface potential and the wave function, a set of coupled equations for the outgoing waves is obtained. The system treats each scattering event as a separate channel, described by a surface **G**-vector, where channels describing measurable peaks are “open”. Though kinematically forbidden and thus not directly accessible, evanescent or “closed” channels receive significant scattering contributions and their consideration is important for numerical convergence. Thus the wavefunctions are numerically solved for in the CC-algorithm, for a finite set of these channels.^42,43^ Appendix B outlines the number of channels considered as well as the integration boundaries handed to the algorithm. The method of solving the equations has been widely discussed in previous publications,^22,42–46^ and is thus not described in the following. Before comparison with measured data, the elastic CC-calculations further need to be corrected for the Debye–Waller attenuation, using an experimentally determined Debye–Waller factor 2*W*.

#### The model interaction potential

As a starting point a corrugated Morse potential^21^ was used, with the leading terms of the interaction potential and its coupling terms *V*_**G**_ for the **G**-vectors **G** ≠ 0 given by:1
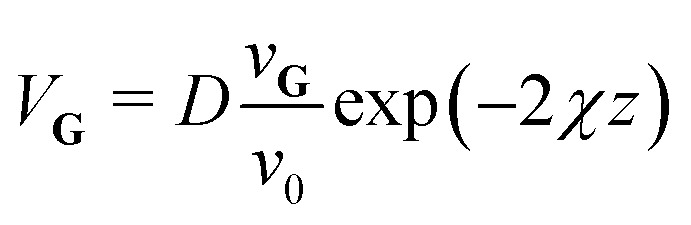
where *D* is the potential well depth, *χ* the potential stiffness and *ν*_0_ is the surface average over exp(−2*χz*). The coefficients *v*_**G**_ are then expressed *via*:2
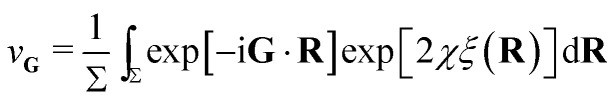
where *Σ* denotes the area of the surface unit cell and *ξ*(**R**) the corrugation function, *i.e.* the classical turning point for the given surface potential. In principle, the coupling terms determine the fraction of the incident beam which is scattered into diffraction channels and thus the scattered intensities. One can see that this is largely governed by the electronic corrugation described by *ξ*(**R**).^21^

In this work, a one-dimensional simplification of the CC-formalism was applied, for the calculation of several diffraction scans along the 
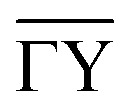
-direction, in order to make the problem computationally tractable. Following the ideas from Miret-Artes *et al.*,^32^ the corrugation function of a stepped or vicinal surface, can be modelled according to:3
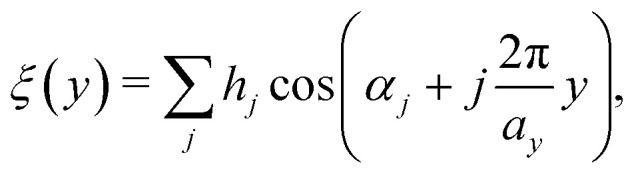
with *a*_*y*_ being the lattice constant in the 
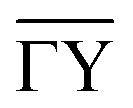
-direction.

Fig. 1(b) shows such a function with *j* = 3 terms, *h* = [0.435,0.43,0.14] and 
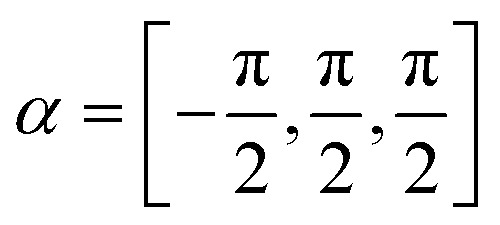
 above the 
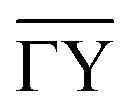
-direction of the Bi(114) surface. It can be seen that three terms are sufficient to reproduce the overall asymmetry of this particular surface, including the protrusion by the atomic row at approximately half the distance of the main modulation.

Treating terms with *j* > 1 as perturbation terms and inserting the resulting expression into eqn (2) yields:4
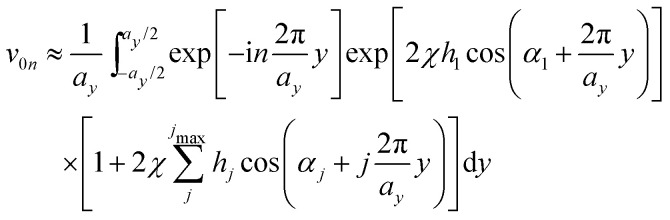
Now, using the following relations (adapted from ref. 47) the exponentials in eqn (4) can be expanded as Bessel functions (*J*_*k*_(*z*)) and modified Bessel functions (*I*_*k*_(*z*)) of integer order:5a
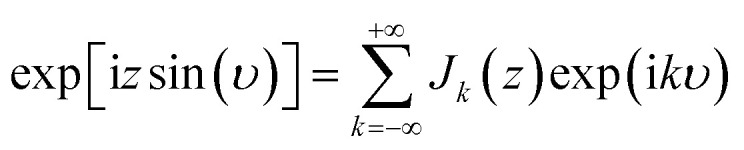
5b
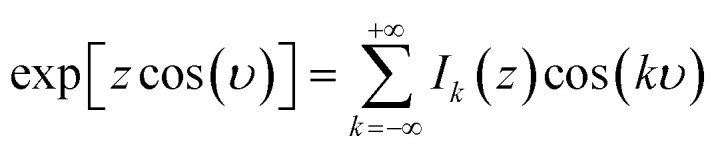
5c
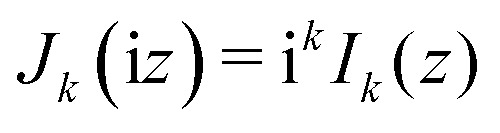
Inserting these relations into eqn (4), one eventually arrives at the following expression for the coefficients *v*_**G**_:6
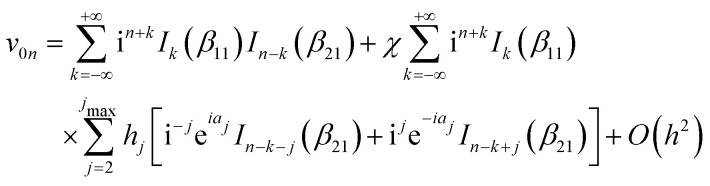
with the arguments of *I*: *β*_11_ = −2*χ*cos(*h*_1_) and *β*_21_ = 2*χ*sin(*h*_1_).

## 3 Results

A multitude of elastic diffraction scans upon scattering normal to the steps such as in Fig. 2(b) was recorded, with varying incident beam energy *E*_*i*_. These angular scattering distributions measured *via ϑ*-scans along the 
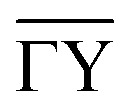
-direction of the sample, are then further analysed based on quantum-mechanical scattering calculations, employing the CC-algorithm.

**Fig. 3:**
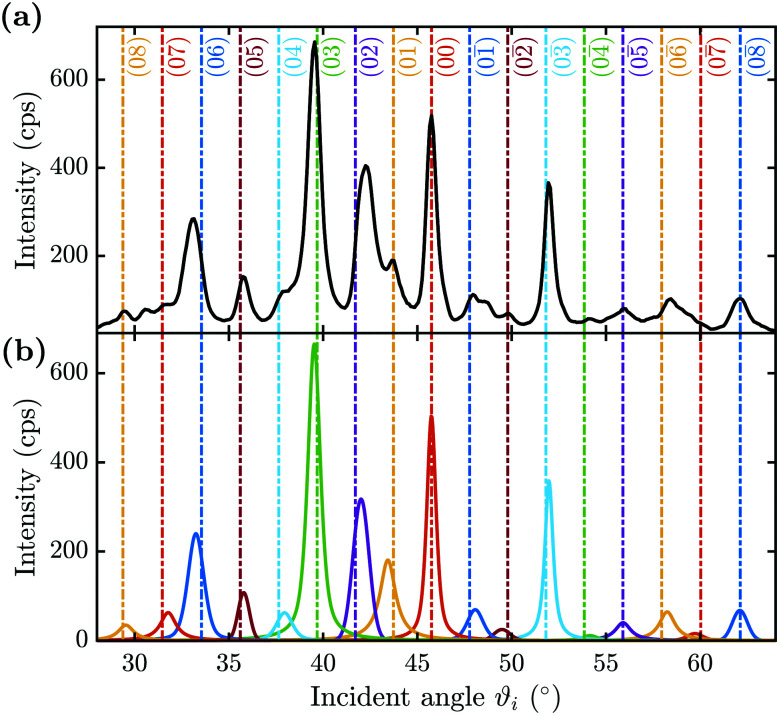
(a) Characteristic *ϑ*-scan of the Bi(114) surface along 
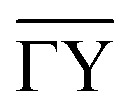
 (measured data shown by the black line) with the sample temperature kept constant at *T*_S_ = 114 K. The energy of the incident helium beam was held constant at *E*_*i*_ = 10.58 meV. The coloured vertical lines represent the theoretically calculated peak positions with the corresponding **G**-vectors. (b) Fits of the measured diffraction peaks using a scaled pseudo-Voigt profile.

Fig. 3(a) shows a typical diffraction scan with the coloured vertical lines denoting the interacting **G**-vector. In Fig. 3(b) the corresponding pseudo-Voigt profiles as the result from a best fit for the overall representation of the above scan, following the curve_fit function from the scipy.optmize package, are plotted. Due to the broadening of the elastic peaks caused by the energy spread of the helium beam and the broadening caused by the apparatus as well as the domain size of the crystal surface, the experimental peak areas, rather than the peak maxima, were used for comparison with the calculated values. Therefore, the peak areas and their respective errors were calculated (see Appendix A for details), normalised to the specular peak and corrected for the Debye–Waller attenuation finally resulting in the experimental intensities *I*_exp_. The Debye–Waller correction was determined *via* the analysis of several elastic measurements of the specular intensity, *I*(*T*_S_), at a fixed incident energy of 11.08 meV and for various sample temperatures *T*_S_.^41,48,49^ The slope *γ* was then extracted from a plot of the natural logarithm of the intensity ln[*I*(*T*_S_)] *versus T*_S_. The Debye–Waller correction was applied *via I*(0) = *I*(*T*_S_)exp[2*W*], with the Debye–Waller factor 2*W* = *γT*_S_ following from the experimentally determined slope *γ* = (−6.0 ± 0.1) × 10^−3^ K^−1^.

Earlier works showed that the position of the rainbow angle in a diffraction scan gives an estimate for the corrugation height of a surface.^50–52^ In fact, a peak-to-peak corrugation of about 0.5 Å yields a rainbow angle of 6.2° and would thus be consistent with the high intensities of the (03) diffraction peaks in Fig. 3(a). However, the simple assumptions made above do neither hold for the herein used scattering geometry nor the asymmetry of the surface corrugation. Moreover, such an analysis, based on a hard wall assumption, does not reflect a realistic atom-surface potential and cannot account for any beam energy dependence of the corrugation. Therefore, we employ quantum mechanical scattering calculations as outlined in the following.

### 3.1 Surface electronic corrugation

To reproduce the measured scattering intensities, the aim of the calculations was to find a corrugation function *ξ*(*y*) as described by eqn (3), ultimately resulting in a set of coupling parameters that determine the amount of intensity scattered into each channel. The shape of *ξ*(*y*) is determined by the terms *h*_*j*_ and *α*_*j*_, resulting in six fit parameters *h*_1–3_ and *α*_1–3_. To avoid additional fit parameters,^32^ values for *D* and *χ* were fixed at 7.898 meV and 0.884 Å^−1^ respectively, as found by Kraus *et al.*^46^ for the Bi(111) surface. Since these values are largely governed by the polarisability of the surface atoms, these should therefore not differ greatly depending on the surface plane. Additionally, since the leading term of the *v*_**G**_ coefficients can always be defined as real, by a change in origin^53^ which in this case is defined by *α*_1_, its value was fixed to 
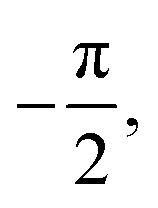
 further reducing the optimisation problem to five fit parameters. Minimisation routines, as described in Appendix B, were then employed to find the optimal set of *h*_*j*_ and *α*_*j*_.

Four diffraction scans, at incident energies of 10.58, 11.17, 11.71 and 13.22 meV, measured for a cooled sample (sample temperature *T*_S_ ≈ 113 K) were analysed. Diffraction scans at higher incident energies were also recorded, however they showed a significant decrease in resolution and the returned peak fits were unsuitable. Furthermore, not all diffraction intensities of a single scan were handed to the calculations, on the one hand to keep the computational time reasonable and on the other hand because not all experimental peaks showed an adequate fit, partly caused by the experimental uncertainties/resolution.

**Fig. 4:**
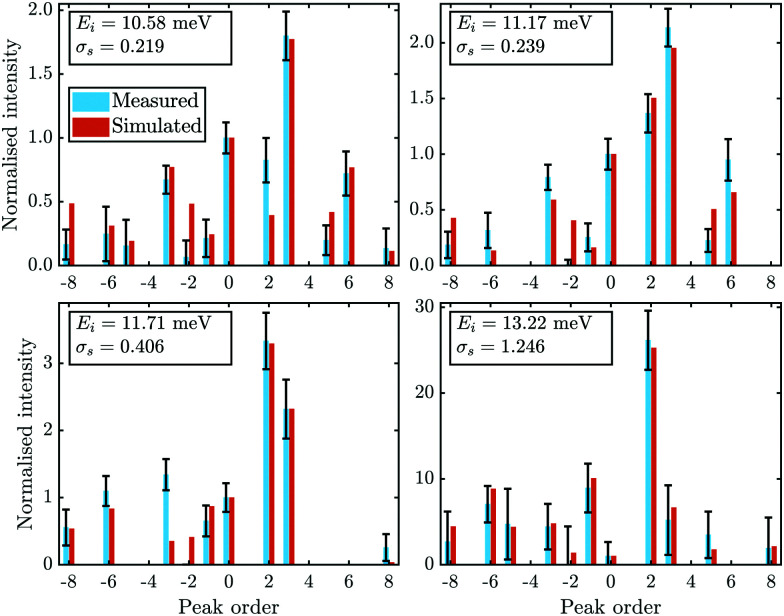
Comparison of the measured (blue bars, see text for details) and the simulated peak intensities (orange bars) for the diffraction peaks along 
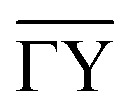
 handed to the optimisation routines (see Fig. 5 for comparison). The error bars of the measurements were calculated with eqn (9).

Fig. 4 compares the experimental intensities of four scans (with increasing beam enery *E*_*i*_) with the calculated diffraction intensities for the optimised corrugation functions, as well as the respective deviation between simulation and experiment *σ*_s_ which was calculated *via*:7
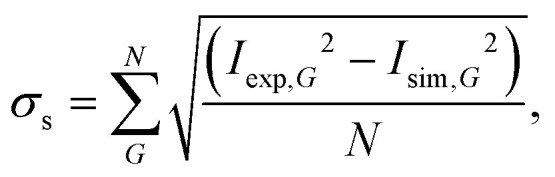
where *G* is the diffraction channel index and *I*_sim_ are the simulated peak intensities. *N* is the number of diffraction peaks in the scan, omitting the specular peak since due to the normalisation its area is set to 1 in both *I*_exp_ and *I*_sim_. Note that *σ*_s_ served as the objective function for the Nelder-Mead minimisation routine (see Appendix B for details)

The best fit values of *h*_*j*_ and *α*_*j*_ for the four scans are given in Table 1. No uncertainties are presented at this point, as the values in Table 1 are simply the values of the minimum found by the routines. However, it was found that varying *h*_1_ and *h*_2_ in the 10^−4^ order of magnitude resulted in an increase of *σ*_s_ by approximately 0.001. The minimum appeared to be more stable towards changes of *h*_3_, *α*_2_ and *α*_3_. Using these values, the experimental peak intensities are well reproduced and most of the simulated intensities are within the experimental uncertainties.

**Table 1:** Values for the parameters *h*_*j*_ and *α*_*j*_ of the corrugation function (eqn (3)) as found by the minimisation routines. Results for *σ*_s_ are also shown (values rounded to three decimal places)

*E*_*i*_/meV	*h*_1_/Å	*h*_2_/Å	*h*_3_/Å	*α*_2_/rad	*α*_3_/rad	*σ*_s_
10.58	0.197	0.070	0.031	1.678	2.692	0.219
11.17	0.190	0.073	0.031	1.557	3.246	0.239
11.71	0.171	0.087	0.014	1.855	3.737	0.406
13.22	0.262	0.063	0.041	2.547	2.745	1.246

**Fig. 5:**
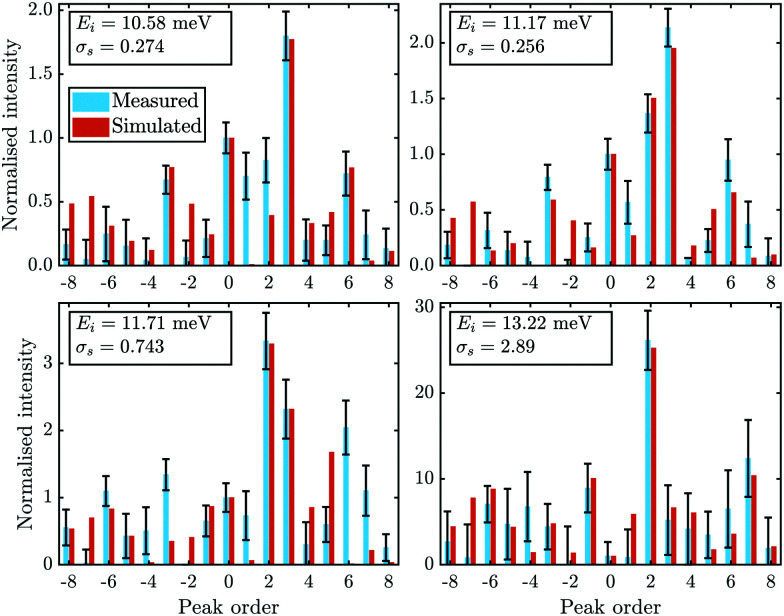
Comparison of all measured intensities (blue bars, *i.e.* including also the ones which where not handed over to the optimisation routines due to their uncertainties) with the simulated peak intensities (orange bars) along 
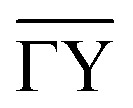
. The error bars for the measurements were calculated with eqn (9). The error bars for the peaks of orders −7 and 4, of the scan with *E*_*i*_ = 11.17 meV were omitted as they showed relative errors 
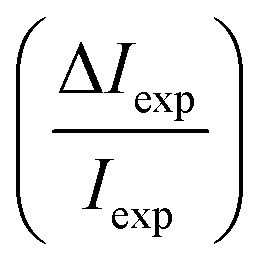
 of 9.5 × 10^6^ and 9.2 × 10^2^ respectively.

In addition to the diffraction peaks handed over to the optimisation routine, Fig. 5 shows a comparison where all peaks have been calculated with the respective corrugation functions obtained above, illustrating that the scattering distributions are also well reproduced in their entirety. One should note that the resolution of the experimental scans decreased with increasing incident energy, which can also be seen by the overall increasing size of the error bars in Fig. 4 and 5. In comparison, the arithmetic mean, 
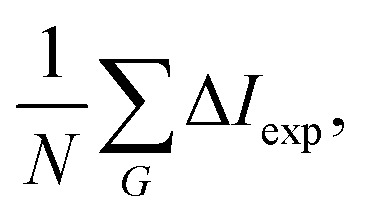
 of the experimental uncertainties ranged from 0.15–3.5 thus explaining the overall increase of the deviation between simulation and experiment in terms of *σ*_s_(7).

**Fig. 6:**
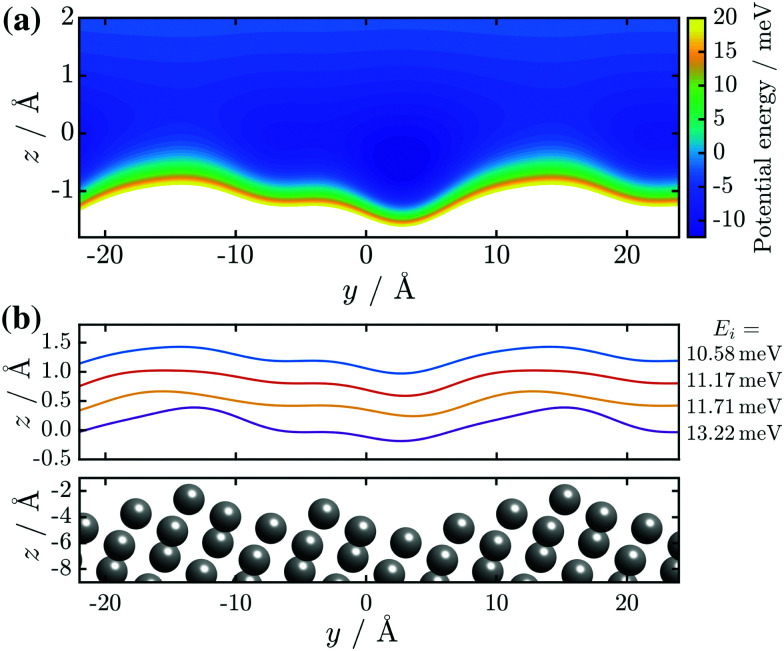
(a) Contour plot of the interaction potential, *i.e.* the potential energy for the best-fit corrugation function as obtained for the lowest beam energy *E*_*i*_ = 10.58 meV. (b) Results of the best-fit corrugation profiles *ξ*(*y*) as calculated with eqn (3) for four diffraction scans with various incident energies *E*_*i*_. A consecutive offset of 0.4 Å along *z* was added to each profile to make them easily distinguishable, and thus the *z*-position of the corrugation functions is not a representation of the atoms' closest approach.

The corresponding corrugation profiles as a result of the optimal set of *h*_*j*_ and *α*_*j*_ are displayed in Fig. 6. Visually, the corrugation functions show little deviation from one another, with the exception of the one found for the highest incident energy (purple line in Fig. 6). The latter may be due to a closer approach of the incident He atoms to the ion cores, with increasing beam energy. The overall shape of the surface profile is also well reproduced. Not only can the overall asymmetry be seen clearly but also the aforementioned protrusion is visible in all of the *ξ*(*y*), due to the value of *h*_2_ in combination with *α*_2_, which places it on the same lateral position for all four functions. The corresponding peak-to-peak corrugation height *ξ*_pp_ for the three lower incident energies is *ξ*_pp_ = 0.46 Å while for *E*_*i*_ = 13.22 meV the value increases to *ξ*_pp_ = 0.6 Å.

Analysis on a Bi(111) surface showed peak-to-peak values of 0.21 Å,^46^ in good agreement when considering that the (114) surface has a much higher corrugation in terms of the ion cores. However, the electronic corrugation profiles as “seen” in HAS experiments on the Bi(114) show clearly a Smoluchowski smoothing, which is also reflected by the topography from scanning tunnelling microscopy (STM) by Wells *et al.*^20^ which shows a corrugation of ≈3.3 Å. The latter is also supported by comparison with the analysis of vicinal copper surfaces, as reported by Miret-Artes *et al.* which resulted in peak-to-peak values of ≈0.5 Å.^32^

### 3.2 Verifying the scattering direction

**Fig. 7:**
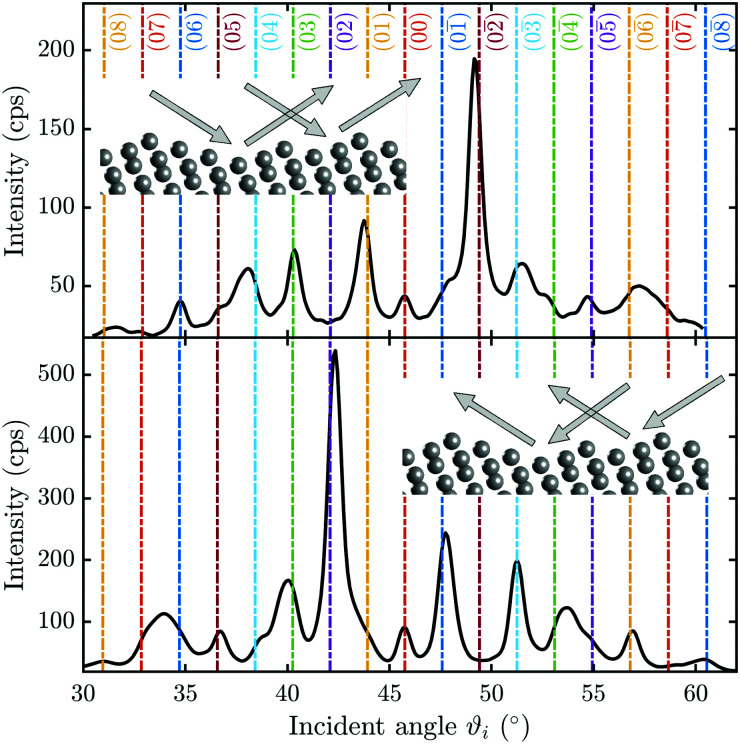
Comparison between two angular diffraction scans along the 
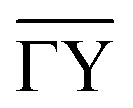
-azimuth: by changing the azimuthal orientation of the crystal *via* a rotation of 180°, the scattering distribution becomes mirrored around the specular peak at *ϑ*_*i*_ = 45.75°. The top panel shows a *ϑ*-scan at room temperature of the rotated sample with *E*_*i*_ = 13 meV while the lower panel, for comparison, shows a *ϑ*-scan at room temperature and *E*_*i*_ = 12.87 meV with the same azimuthal orientation as in the prior analysis.

From the experimental data alone, the orientation of the in- and outgoing beam with respect to the stepped surface structure, is unknown. As shown by the insets in Fig. 7, upon scattering normal to the steps, the impinging helium atom “sees” a different electronic corrugation, depending on the azimuthal rotation of the crystal. To clarify this issue, quantum-mechanical scattering calculations can be utilised to determine the orientation of the steps with respect to the incoming beam and thus the azimuthal rotation of the crystal.

Fig. 7 shows two *ϑ*-scans with the sample at room temperature, where the azimuthal orientation of the crystal is rotated by 180° with respect to each other. From Fig. 7 it becomes evident that the peak height distribution is mirrored around the specular peak (*ϑ*_*i*_ = 45.75°), as expected. However, due to the Debye–Waller attenuation the intensities at *T*_S_ = 300 K are much smaller than for the cooled scans used above, which resulted in difficulties when fitting the pseudo-Voigt profiles to the diffraction peaks. Therefore, the simulations were carried out for a cooled scan with *E*_*i*_ = 10.58 meV, where the mirroring of the angular intensity distribution around the specular peak was done artificially, by simply changing the sign of the interacting **G**-vectors from here on called quasi-mirrored scan.

It was found that shifting *α*_2_ by 
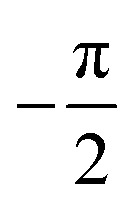
 shifts the protrusion and asymmetry in *ξ*(*y*), resulting in a mirrored unit cell along *y* in Fig. 1(b), *i.e.* an azimuthal rotation by 180°. Therefore, the Nelder Mead routine (see section Appendix B) was run for this quasi-mirrored scan where the initial values of *h*_*j*_ and *α*_*j*_ were set to the optimal values found for that particular scan, but with *α*_2_ shifted by 
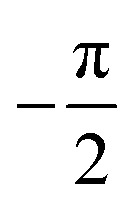
.

**Fig. 8:**
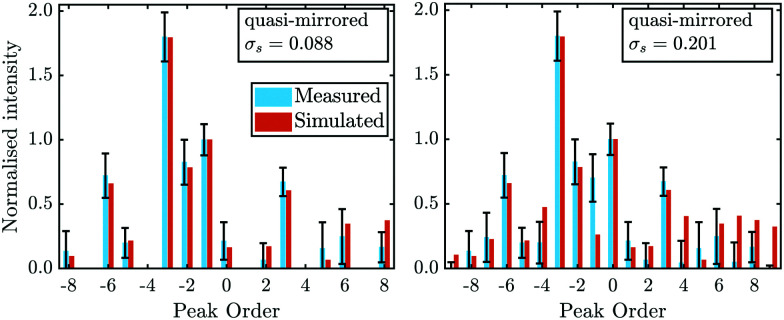
Comparison of the measured (blue) and simulated (orange) peak intensities for the quasi-mirrored scan with *E*_*i*_ = 10.58 meV. The left graph shows only the calculated intensities for the ones handed to the optimisation routine, while in the right graph all calculated peak intensities are plotted.

The results from the scattering calculations are plotted in Fig. 8, illustrating that the peak intensity distribution of the quasi-mirrored scan is reproduced rather well with a negative *α*_2_. The corresponding parameters of the corrugation function are given in Table 2.

**Table 2:** Values for the parameters *h*_*j*_ and *α*_*j*_ of the corrugation function (eqn (3)) as found by the minimisation routines for the quasi-mirrored scan scan (rounded to four decimal places)

	*h*_1_/Å	*h*_2_/Å	*h*_3_/Å	*α*_2_/rad	*α*_3_/rad
Quasi-mirrored	0.221	0.093	0.027	-2.044	2.416

To rule out a coincidental match, the un-mirrored scan was simulated again with the now negative value of *α*_2_, which clearly cannot reproduce the peak distribution of the former diffraction scan. It suggests that the method is in fact capable of determining the azimuthal rotation of the crystal, *i.e.* the orientation of the steps with respect to the incoming beam. The classical blazed optical reflection grating shows in fact the same property by shifting the maximum intensity from the positive nth order to the negative nth order upon changing the step inclination to its mirror image.^36^

For a firm validation scans with the mirrored sample, with the same or at least similar conditions to those in the previous section, should be analysed and compared. Another interesting qualitative comparison with an optical reflection grating arises when the step inclination is estimated which would be necessary to arrive at a maximum diffraction order for the second and third orders for wavelengths between 1.15 to 1.25 Å. The corresponding angle with respect to the grating plane should be around 4°, not far from the averaged angle obtained for the lattice corrugation of 3.3 Å and the step length of 28.4 Å.

## 4 Conclusion and outlook

Vicinal surfaces provide an attractive path to study systems of reduced dimensionality and here we have reported a study of the quasi one-dimensional topological metal Bi(114), based on atom-surface scattering experiments. In general, diffraction scans of “asymmetrically” stepped surfaces in combination with the large unit cell, make both a peak separation experimentally difficult and the analysis challenging. We have provided a foundation for more accurate representations of atom-surface interaction potentials and the surface electronic corrugation of such vicinal surfaces.

As outlined, the surface electronic corrugation of Bi(114) upon scattering normal to the steps, can be determined together with quantum mechanical scattering calculations. By varying the shape of the corrugation function, elastic scattering measurements performed on a Bi(114) surface are reproduced to a good degree of accuracy. The resulting corrugation profiles, determined as the best fit from minimisation routines, show that the atomic asymmetry along the 
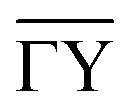
-direction of the sample is also represented in the surface electronic corrugation. The latter is further underlined by demonstrating that it is in principle possible, to determine the orientation of the in- and outgoing beam with respect to the stepped surface structure, based on scattering calculations. The peak-to-peak amplitudes of the determined surface electronic corrugation profiles are larger than compared to the low-index Bi(111) surface, but are still much smoother compared to the position of the ion cores, as also additionally reflected in comparison with the topography from STM measurements.

It should also be noted, that in order to develop the full He–Bi(114) interaction potential other potential parameters of the potential, such as the well depth and its stiffness need to be considered. It cannot be said with certainty that the (114) surface exhibits the same parameters as the (111) surface of bismuth as studies have shown that they may in fact vary depending on the surface plane.^54^ A precise experimental determination of these parameters would therefore be of interest in order to further develop and refine the interaction potential. Finally, for a better representation of the surface one could also consider constructing a three dimensional corrugation function and calculating two dimensional coupling terms and coefficients in the form *v*_*mn*_. This would allow for the consideration of scattering contributions into out of plane channels and therefore for more accurate simulations of the experiments.

## Author contributions

S. J. S. performed the quantum mechanical scattering calculations and carried out most of the data analysis. A. T. and A. R. performed the experimental measurements while S. J. S., A. R. and A. T. developed the physical interpretation of the data. All authors discussed the results and contributed to writing the manuscript.

## Data and code availability

The datasets generated and analysed during the current study are available from the TU Graz repository, with the identifier https://doi.org/10.3217/yetw4-ahr29. An implementation of the CC algorithm is available from https://repository.tugraz.at/records/cd0y0-xa478 under the GNU General Public License v3.0.

## Conflicts of interest

There are no conflicts to declare.

## Supplementary Material

## Appendices

### A Experimental peak areas

The peak areas were calculated by fitting a scaled pseudo-Voigt profile to the measured data, using the curve_fite function from the scipy.optmize package. The profile is a combination of a Gaussian and a Lorentzian profile which are mixed according to a mixing parameter *η*. The area *A* of such a profile is calculated *via*:

8

where *I*_0_ is the peak amplitude and 2*ω* the full width at half maximum. The curve_fite function also returns a covariance matrix with which the standard deviations *s* of the individual fit parameters can be calculated. The uncertainty of the areas *σ*_A_ can then be determined with:

9

where *s*_*I*_0__, *s*_*ω*_ and *s*_*η*_ are the standard deviations of the estimates for the amplitude, the width and the mixing parameter respectively.

### B Minimisation routines

The first major routine to be discussed is the Differential Evolution (DE) algorithm, based on the ideas of Storn and Price.^55^ This is a stochastic global optimisation routine, which, depending on the test function is well suited for minimisation of non-linear and non-differentiable continuous space functions.^55^ In this case, the objective function was defined as the absolute difference/error between the measured and simulated peak intensities:

10

The algorithm begins by initialising a population of size *NP* vectors, each consisting of uniformly randomly generated values within previously defined bounds. What follows are steps for mutation and crossover, for which the *rand/1/bin* method is employed. Here the mutant vector *x*_mut_ is created by perturbing a randomly selected vector *x*_*r*1_ with *1* weighted difference of two other vectors *x*_*r*2_ and *x*_*r*3_:

11 *x*_mut_ = *x*_*r*1_ + *F* × (*x*_*r*2_ − *x*_*r*3_)

where *F* is the weighting factor or mutation constant. Crossover then mixes the information of a previously determined target vector (the vector with the lowest objective function value) and the mutant, here *via* binomial crossover (hence *rand/1/bin*). A crossover probability *CR* is used to determine whether a value of the target vector should be replaced by a value of the mutant. The resulting vector then becomes the new target vector if its objective function value is lower. This scheme is repeated for each vector in the population, denoting one iteration of the DE algorithm. After several iterations, the population should converge to the global minimum of the objective function. The outcome is thus largely governed by the control variables NP, *F* and CR.

Following the rules for the usage of DE as formulated by Storn (see ref. 56) the absolute error was used rather than the sum of error squares as the objective function, since the latter has the potential to hide the path to the global minimum.^56^ Since the absolute error can however yield more local minima, larger values of *F* = 0.8 and CR = 0.7 were chosen to increase the search radius and the chance of “leaving” a local minimum.

Bounds for the search in *h*_*j*_ and *α*_*j*_ were also introduced and set to:*h*_1_ = [0,0.435], *h*_2_ = [0,0.43], *h*_3_ = [0,0.14]

While it may seem arbitrary, the bounds were chosen by comparing the resulting corrugation function *ξ*(*y*) to the atomic representation as seen in Fig. 1. The bounds for *α*_2_ were set to the above values, since these cover the functional shapes of possible corrugation profiles for both  scattering directions.

The upper bounds of *h*_*j*_ were chosen as above, to ensure that even very extreme corrugation profiles would be searched, and additionally to guarantee that convergence could still be reached with a reasonable number of closed channels. Thus, for the DE routine, all open channels together with 165 closed channels were used for the calculations, with the integration bounds along the *z*-direction set to [−6,8] Å and with 28 steps per minimum observed scattering channel wavelength.

As it is often the case, a so called polishing routine was then used for the first set of optimal parameters found by the DE algorithm. For this the Nelder-Mead method, as part of the scipy.optmize package, was applied. The algorithm as described by Nelder and Mead (see ref. 57) uses a simplex, which is a *N* + 1 dimensional construct in the space of the *N*-dimensional objective function. Each vertex of the simplex thus constitutes a set of values for the fit parameters. It then adapts itself to the local landscape, elongating down long inclined planes, changing direction on encountering a valley at an angle, and contracting in the neighbourhood of a minimum.^57^ The routine was then fed the optimal values as found by the DE algorithm as initial values, to give rise to a further improvement. Furthermore, the more conventional *σ*_s_, as described in the main text, was used as the objective function. The next step was to use the improved values as starting points for the next scans. As for the number of channels and integration parameters, they were chosen according to the corrugation amplitude resulting from the DE routine. They were ultimately set to 50 closed channels, *z* = [−8,9] Å and 35 steps per minimum wavelength for all of the scans that were analysed.
